# Adipogenesis in Different Body Depots and Tumor Development

**DOI:** 10.3389/fcell.2020.571648

**Published:** 2020-09-22

**Authors:** Drenka Trivanović, Sanja Vignjević Petrinović, Ivana Okić Djordjević, Tamara Kukolj, Diana Bugarski, Aleksandra Jauković

**Affiliations:** ^1^IZKF Group Tissue Regeneration in Musculoskeletal Diseases, University Clinics, Wuerzburg, Germany; ^2^Bernhard-Heine Center for Locomotion Research, University of Wuerzburg, Wuerzburg, Germany; ^3^Laboratory for Neuroendocrinology, Institute for Medical Research, University of Belgrade, Belgrade, Serbia; ^4^Laboratory for Experimental Hematology and Stem Cells, Institute for Medical Research, University of Belgrade, Belgrade, Serbia

**Keywords:** adipose tissue depots, adipogenesis, trans-differentiation, tumor, microenvironment, adipose tissue remodeling, stem cell niche

## Abstract

Adipose tissue (AT) forms depots at different anatomical locations throughout the body, being in subcutaneous and visceral regions, as well as the bone marrow. These ATs differ in the adipocyte functional profile, their insulin sensitivity, adipokines’ production, lipolysis, and response to pathologic conditions. Despite the recent advances in lineage tracing, which have demonstrated that individual adipose depots are composed of adipocytes derived from distinct progenitor populations, the cellular and molecular dissection of the adipose clonogenic stem cell niche is still a great challenge. Additional complexity in AT regulation is associated with tumor-induced changes that affect adipocyte phenotype. As an integrative unit of cell differentiation, AT microenvironment regulates various phenotype outcomes of differentiating adipogenic lineages, which consequently may contribute to the neoplastic phenotype manifestations. Particularly interesting is the capacity of AT to impose and support the aberrant potency of stem cells that accompanies tumor development. In this review, we summarize the current findings on the communication between adipocytes and their progenitors with tumor cells, pointing out to the co-existence of healthy and neoplastic stem cell niches developed during tumor evolution. We also discuss tumor-induced adaptations in mature adipocytes and the involvement of alternative differentiation programs.

## Introduction

Adipose tissue (AT) is a loose connective tissue responsible for lipid storage, which also serves as an important protector from excess levels of lipids and glucose (Abate, 2012). Scattered throughout the body, without uniform organization and anatomy, different AT compartments participate in systemic and regional control of the overall energy metabolism and signaling, while their secretomes act locally (paracrine function) and systemically (endocrine function).

The functional classification of AT recognizes energy-storing white adipocytes as the predominant cell type of white adipose tissue (WAT) and heat-dissipating brown adipocytes in brown adipose tissue (BAT) with distinct cellular organizations and metabolic and endocrine functions. However, the delineation of adipocyte phenotypes is not strict, since brown adipocytes were found sporadically in the WAT of mice (Lucchini et al., 2020). Also, an intermediate adipocyte profile characterizes beige or “brite” adipocytes, which represent an inducible form of thermogenic adipocytes sporadically residing within WAT (Bukowska et al., 2018; Schoettl et al., 2018). Although both WAT and BAT adipocytes can store large amounts of lipids, their main functional differences relate to the higher oxidative capacity and energy expenditure by BAT under nervous/endocrine control (Alemany, 2011). While BAT depots are primarily found in rodent and human infants, their increased activity in adults can be demonstrated upon cold exposure, noradrenergic and pharmacological stimulation, or fasting.

Depending on their anatomical location, adipose depots are broadly classified into the subcutaneous, intramuscular, visceral, and bone marrow subtypes (Vaitkus and Celi, 2017). In humans, subcutaneous adipose tissue depots (SAT) serve as entity of energy storage and protection against mechanical damage and heat loss (Alexander et al., 2015; Kasza et al., 2019). Visceral adipose tissue (VAT) stores abdominal triglycerides forming a coating layer over the vital organs in the omentum and the retroperitoneal space. Bone marrow adipose tissue (BMAT), also referred to as yellow AT, is another, distinct AT subtype inhabiting the rigid bone cavity, which regulates hematopoiesis, the skeletal system, paracrine/endocrine signaling, and energy metabolism (Suchacki and Cawthorn, 2018).

Both classifications of AT reflect its heterogeneity at a cellular level and anatomical localizations, thus compromising the understanding functional differences between adipocytes and their associations with the development of various diseases. This review summarizes the data on the AT microenvironment at different body depots and its remodeling during tumor development.

### Adipose Tissue Niche Constituents

In addition to adipocytes, the complex biological entity of the AT niche includes endothelial cells, macrophages, and other immune cells and a small size population of stem cells within adipose stromal cells (ASCs) (Kim et al., 2014). AT homeostasis and remodeling are regulated by the interactions of these cells residing within a specific milieu of secreted factors and an extracellular matrix (ECM). Depending on the depot location, health status, age, and gender, variations were found in cellular composition, the ASC compartments, vascularity, the content of the ECM, metabolism, and the secretome of the AT niche (Lee et al., 2013). While large, insulin-resistant adipocytes, which are more sensitive to lipolysis, characterize VAT with a rich vasculature and innervation, containing higher levels of macrophages, T cells, and natural killer cells, SAT is characterized by small insulin-sensitive adipocytes, lower vascularity, innervation, and immune cell infiltration (Ibrahim, 2010). Compared with the other AT depots, BMAT displays distinctive features as a more heterogeneous depot containing adipocytes of divergent phenotypes (Hardouin et al., 2016) that display a specific cholesterol-orientated lipid metabolism devoid of lipolytic activity (Attané et al., 2020).

The functional differences of AT depots result from the heterogeneity of ASCs influenced by specific depot microenvironments. The lobules as structural units within human AT depots are composed of two ECM compartments, stroma and septa. This was proposed to delineate the niches of progenitor subsets: the stromal vascular fraction (SVF) containing AT progenitors (APs) (CD45^–^CD34^+^CD31^–^ALPL^+^) and the fibrous septa mostly inhabited by myofibroblast progenitors (CD45^–^CD34^+^CD31^–^CD271^high^), which are distributed differently in SAT and VAT (Estève et al., 2019). *De novo* adipogenesis takes place in proximity to the vasculature, particularly in the *tunica media* and *tunica adventitia*, where pericytes and fibroblasts share a similar mesenchymal phenotype with overlapping expressions of PDGFRα (fibroblasts) and PDGFRβ (pericytes) (Jiang et al., 2014; Guimarães-Camboa and Evans, 2017; Sun et al., 2019). Lineage-tracing studies revealed that during development, BAT uncoupling protein 1 (UCP1)-expressing adipocytes derived from *Myf5*^+^ and *Pax7*^+^ cells, while WAT adipocyte developed from PDGFRα- and PDGFRβ-expressing cells (Ikeda et al., 2018). Although cultured stromal vascular fraction (SVF) pericytes displayed multilineage differentiation capacity *in vitro* and *in vivo* (Lin et al., 2008; Rodeheffer et al., 2008), lineage-tracing experiments investigating WAT and BAT of mice showed that pericytes did not behave as APs *in vivo* (Guimarães-Camboa et al., 2017), while fibroblast progenitors undergoing adipogenesis acquired a beige phenotype before their differentiation into mature WAT cells (Cattaneo et al., 2020). In addition to the lower number of APs found in VAT (Tchkonia et al., 2005), a study utilizing the transplantation approach demonstrated that the proliferation and the differentiation of mouse APs (Lin^–^CD29^+^CD34^+^) are influenced by the depot and their context-specific microenvironment, suggesting that APs represent functionally plastic cells (Jeffery et al., 2016). Human abdominal SAT-derived APs were shown to have an increased adipogenesis and growth rate than VAT-derived APs, with a reduced susceptibility to lipolysis, suggesting that adult APs display differences inherent to the AT source (Tchkonia et al., 2006; Gealekman et al., 2011; Baglioni et al., 2012). Single-cell (sc) RNA profiling of mouse VAT-derived SVF identified adipogenic subpopulation Ly6C^–^CD9^–^PDGFRβ^+^ as adipocyte precursors and Ly6C^+^ PDGFRβ^+^ as fibro-inflammatory progenitors, where labeling and tracing of PDGFRβ-expressing cells in VAT allowed deconvolution of these functionally distinct APs (Hepler et al., 2018). Furthermore, analysis of mouse Lin^–^CD29^+^CD34^+^Sca-1^+^ and corresponding population in human AT revealed that a high expression of additional markers, CD142 and ABCG1 (cholesterol transporter), are associated with the presence of APs with decreased adipogenic potential, the so-called regulatory adipocytes (Schwalie et al., 2018). Thus, hierarchical organization of ASCs implies the existence of specialized progenitor entities involved in regulation of adipogenesis and inflammation, bringing an additional complexity to the adipogenic progenitor niche.

On the one hand, marrow adipocyte develops from bone marrow mesenchymal stromal cells (BM-MSCs), where their precursors share the features of osteoblasts, expressing leptin receptors (Zhou et al., 2014), osterix (Mizoguchi et al., 2014), and nestin, as described in animal models (Horowitz et al., 2017). On the other hand, human BM-MSCs *in vitro* did not recapitulate marrow adipogenesis or the functional properties of BMAT, such as reduced lipolysis (compared with SAT) and a cholesterol-based metabolism (Scheller et al., 2016; Attané et al., 2020). The phenotype of the BMAT progenitors is still unknown, while a recent study based on large-scale scRNA-sequencing data indicated the existence of nonproliferative marrow APs in mice, which are responsible for BMAT formation (Zhong et al., 2020).

Nevertheless, the heterogeneity of different AP phenotypes, their overlapping features, and their relations to cellular functionality remain to be elucidated. Certainly, detailed insights into the architecture of AT depots, their cellular composition, and secretome will improve the understanding of each AT depot microenvironment in its specificity.

### The Course of Adipogenesis and the Dynamic (Inter) Conversion of the Adipocyte Phenotype

Although the pool of adipocytes is set during childhood and remains constant through adulthood, it may be increased upon early-onset overnutrition, through obesity, or even caloric restriction in the case of BMAT (Abate, 2012; Cawthorn et al., 2016). The increase in AT mass can occur through adipocyte hypertrophy (lipid-accumulation-induced enlargement) and hyperplasia (the proliferation/differentiation of stem cells/preadipocytes resulting in increased adipocyte numbers), and these processes are followed by chronic tissue inflammation and fibrosis (Hepler et al., 2018; Kahn et al., 2019). Learning from *in vitro* and *in vivo* rodent models, adipogenic differentiation involves a set of gradual stages including stem cells/preadipocyte growth arrest, mitotic clonal expansion, terminal differentiation, and maturation (Tang and Lane, 2012; Chen et al., 2017). Even though the mechanisms underlying adipogenic differentiation are not fully elucidated, the importance of the transcriptional regulator Zfp423 (Gupta et al., 2012) and balance of PDGFRα/PDGFRβ signaling in the regulation of progenitor commitment to beige (PDGFRα) or white (PDGFRβ) adipocytes has been demonstrated (Gao et al., 2018). Moreover, during adipogenesis, sequential transcriptional regulation in preadipocytes by peroxisome proliferator-activated receptor-γ (PPARγ), CCAAT-enhancer-binding proteins, and PPARγ coactivator 1-α, guides the mitochondrial biogenesis and increased oxygen consumption rate of the differentiated adipocytes (Lee et al., 2019).

**FIGURE 1:**
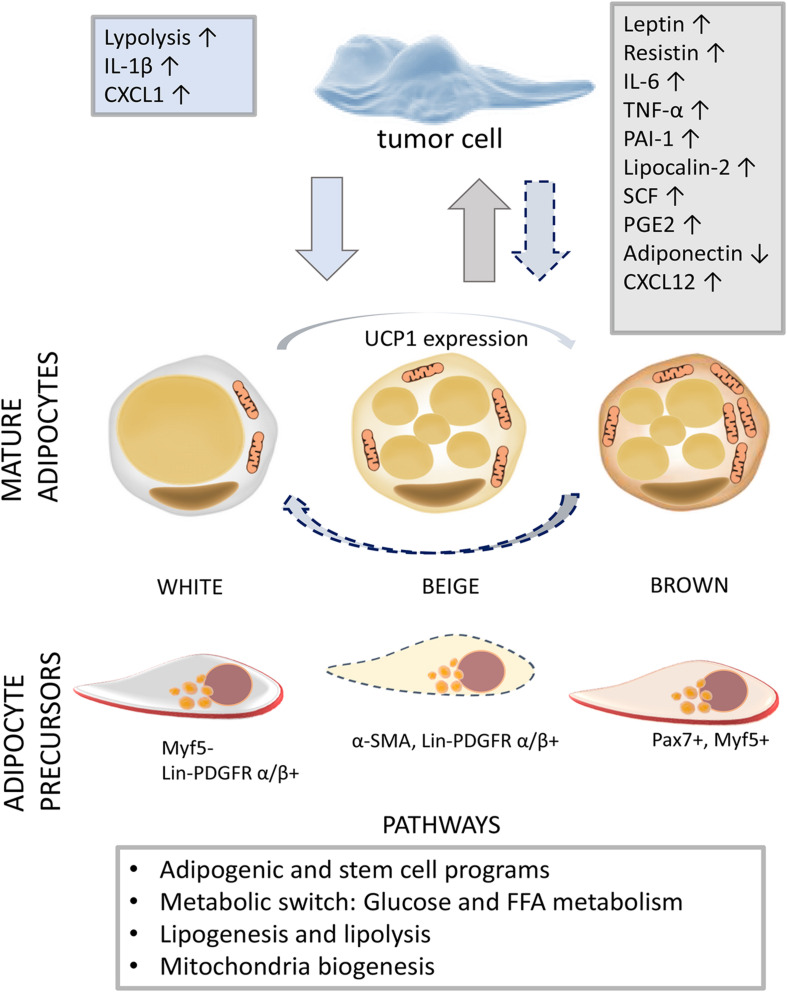
The diversity of adipogenesis in a tumor milieu. The activation of different adipogenic programs in adipose tissue (AT) and tumors reveals the capacity of adipogenic populations and neoplastic cells for phenotype conversion. In a tumor microenvironment, associated with low-grade inflammation, mature adipocytes and resident APs adjust their biochemical profile (white to brown and brown to white) and commitment, respectively. The dashed arrow indicates the development of tumor-supportive AT cells in response to tumor-derived factors. On the other hand, tumor cells can acquire adipogenic features, providing itself nutrition, survival, and further development through mainly unknown intermediate phenotype states. Adipokines (leptin, resistin, PAI-1, lipocalin-2, and adiponectin), cytokines (IL-6, TNF-α, and SCF), and chemokines (CXCL12) secreted by AT cells are indicated in the gray box, while tumor-derived factors are written in the blue box (Lengyel et al., 2018). Abbreviations: IL, interleukin; CXCL, C-X-C motif chemokine ligand; TNF-α, tumor necrosis factor alpha; PAI-1, plasminogen activator inhibitor-1; SCF, stem cell factor; PGE2, prostaglandin E2; UCP1, uncoupling protein 1; Myf5, myogenic factor 5; α-SMA, alpha-smooth muscle actin; Lin, lineage; PDGFR, platelet-derived growth factor receptor; Pax7, paired box protein 7; AT, adipose tissue; AP, AT progenitor.

Mitochondria are key organelles controlling adipocyte differentiation, lipid homeostasis, insulin responsiveness, oxidative capacity, and adaptive thermogenesis in adipocytes (Sanchez-Gurmaches et al., 2016; Lee et al., 2019). All these processes appeared to be involved in dynamic phenotype conversion, which is characteristic of WAT browning and BAT whitening. WAT adipocytes have fewer, elongated mitochondria selectively expressing proteins that support lipogenesis and degrade xenobiotics. Compared with WAT, BAT adipocytes possess larger mitochondria featured by a higher expression level of UCP1, fatty acid oxidation-related enzymes such as acetyl-CoA dehydrogenase and catabolic metabolism (Diedrich et al., 2018; Lee et al., 2019). The thermogenic capacity marker, UCP1, functions to uncouple the oxidative phosphorylation from ATP synthesis in the inner mitochondrial membrane and dissipate energy in the form of heat. Beige adipocytes are also described as UCP1^+^ cells with a dense mitochondrial network and multilocular lipid droplets (Figure 1). In physiological conditions, beige adipocytes act as white adipocytes, while in response to environmental factors such as cold exposure, exercise, cancer cachexia (CC), and tissue injury, their BAT-like function is induced independently of UCP1 (Diedrich et al., 2018; Ikeda et al., 2018). In contrast, the whitening of mouse BAT adipocytes upon temperature change, leptin receptor deficiency, β-adrenergic signaling inhibition, or lipase deficiency leads to decreased mitochondrial content and increased mitophagy (the autophagic degradation of mitochondria) promoting WAT-like tissue formation (Altshuler-Keylin et al., 2016; Ikeda et al., 2018; Kotzbeck et al., 2018). However, a recent study suggests that warm-induced beige adipocyte whitening is mostly based on morphological changes, while beige adipocytes retain epigenetic memory (Roh et al., 2018). Results obtained by scRNA-sequencing elucidated the activation of CD44 and PDGFRα-expressing WAT progenitors, involved in AT browning, through their proliferation and interaction with macrophages (Burl et al., 2018). These findings provide important insights into the capacity of AT to adopt environmental stimuli and adjust to the physiological context by changing the biochemical properties of mature adipocytes and APs.

## Adiposity Parameters in Tumor Patients

The prognostic significance of adiposity at the point of cancer diagnosis and prognosis is not completely clear. While some studies showed an association between obesity and a poor survival rate (Meyerhardt et al., 2003; Kasenda et al., 2014), others have reported obesity to be associated with lower mortality rates (Martin et al., 2013; Schlesinger et al., 2014; Ebadi et al., 2017). Estimating the VAT index (VATI) and the SAT index (SATI) as adiposity markers, SAT appears to correlate with a reduction in mortality risk for gastrointestinal, respiratory cancer, and renal carcinoma patients (Ebadi et al., 2017) and an increased survival rate among patients with bone metastases, thus demonstrating the prognostic value of AT distribution (Chuang et al., 2020). In colorectal tumor patients, low SATI and VATI are associated with an increased risk of dying, as did high SAT and VAT density, where high VAT density was the main predictor of a poor survival rate in metastatic colorectal tumor patients (Charette et al., 2019). Breast tumor patients with axillary lymph node metastasis had a significantly higher VAT/SAT ratio than the patients without metastasis, suggesting that the VAT/SAT ratio might be a useful biomarker of breast tumor aggressiveness (Pahk et al., 2020). Moreover, breast tumor patients exerted a high prevalence of BAT activity than did patients suffering from other solid neoplasms (Singh et al., 2016). Although marrow adiposity is an important contributor to tumor progression in bones, distinguishing physiologic red marrow re-conversion from marrow disease is still a challenge for imaging-based diagnostics (Navarro et al., 2017). The marrow adipocyte area and diameter in acute myeloid leukemia (AML) patients were shown to be smaller than those in healthy subjects, thus indicating a prognostic value of these parameters (Lu et al., 2018). Collectively, these data indicate that the AT profile at different localizations could be clinically significant in the identification of parameters relevant for tumor diagnosis and potentially its outcome.

### Tumor-Induced Adipose Tissue Remodeling

Through metabolic reprogramming, the production of lipids, and the secretion of hormones and adipokines, AT can affect tumorigenesis, tumor growth, survival (Diedrich et al., 2015), and even chemoresistance (Sheng et al., 2017). The mechanisms underlying the effects of AT on tumor cells are nicely summarized in a number of review articles (Diedrich et al., 2015; Corrêa et al., 2019; Quail and Dannenberg, 2019). However, AT–tumor interactions are reciprocal, as tumors affect surrounding AT cells (mature adipocytes, preadipocytes, or APs), by imposing their needs for the purpose of their own evolution. Adipocytes located in close proximity to the invasive tumor cells [often termed as tumor-associated adipocytes (TAAs)] display a modified phenotype, metabolism, and secretome (Figure 1). In addition to a fibroblast-like phenotype implying their dedifferentiation, TAAs are characterized by increased production of inflammatory cytokines, chemokines, adipokines, and high-energy metabolites (Dirat et al., 2011; Ribeiro et al., 2012; Wu et al., 2019).

Moreover, as in the case of CC, systemic alterations caused by the tumor may affect AT remodeling. CC is a multifactorial syndrome that includes the progressive reduction of body weight, associated with the reduction of adipose (mostly WAT) and skeletal muscle tissues, which is often considered a negative prognostic factor in cancer patients. The tumor-provoked hypermetabolism in CC leads to a state of energy imbalance that cannot be recovered through nutritional supplementation (Kir and Spiegelman, 2016; Vaitkus and Celi, 2017). According to morphologic modifications, decreased lipid content and a proinflammatory phenotype, cachectic adipocytes are similar to TAAs (Rybinska et al., 2020). As recently demonstrated, the downregulation of adipogenic and lipogenic genes is affected early in AT, resulting in lipolysis and AT remodeling during CC (Batista et al., 2013; Sun et al., 2020). Also, CC is associated with fibrosis and inflammatory cell infiltration in SAT (Batista et al., 2016), while VAT shows a low lipolysis rate in the early stage of CC and a low *de novo* lipogenesis rate in later stages. In addition, VAT adipocytes as well as SVF isolated from rats were shown to produce increased levels of IL-1β activating inflammasome pathway, suggesting a proinflammatory phenotype of AT cells in CC (Neves et al., 2016).

Another aspect of tumor-affected adipogenesis is related to the stimulation of the browning process in AT. The increased thermogenic activity of AT contributes to the accelerated energy expenditure and weight loss in mouse models of CC whereby IL-6 and parathyroid hormone-related protein, a tumor-derived small polypeptide that modulates calcium homeostasis, were identified as drivers of AT browning (Kir et al., 2014; Kir and Spiegelman, 2016). Yet evidences on adipocyte browning in CC in humans are still missing. Moreover, the white-to-brown trans-differentiation of omental adipocytes was found in patients affected by pheochromocytoma (Frontini et al., 2013) and breast cancer where mammary tumor stem cells expressed PRDM16, a master regulator of brown adipocyte differentiation (Singh et al., 2016). The promoted beige/brown program also correlates with stimulated adipocyte catabolism characterized by the release of fatty acids, pyruvate, lactate, and ketone bodies supplying tumor cells. Accordingly, hypermetabolic breast tumor cells feature increased glucose uptake and triglyceride accumulation (Kir and Spiegelman, 2016; Wu et al., 2019; Figure 1).

Interestingly, tumor-provoked physical stresses can induce adipocyte dedifferentiation into a state similar to MSCs, termed as the compression-induced dedifferentiation of adipocytes, characterized by a clonogenic and multilineage differentiation capacity *in vitro* (Li et al., 2020). However, this observation needs to be validated in human cancer *in vivo*, considering that ASCs obtained from breast tumor patients display an impaired adipogenic capability (Rey et al., 2019). Besides, human ASCs from the intra-abdominal VAT of prostate cancer patients primed with tumor-derived exosomes can undergo neoplastic transformation exerting cytogenetic aberrations, and a mesenchymal-to-epithelial transition, along with the capacity to form tumors *in vivo* (Abd Elmageed et al., 2014). Importantly, ASCs from periprostatic WAT of prostate cancer patients promote tumor cell epithelial-to-mesenchymal transition and cancer aggressiveness, while their depletion might help chemotherapy efficiency in mice (Su et al., 2019). Moreover, near the tumor cells of breast cancer patients, ASCs (Jotzu et al., 2010) and stromal adipocytes (Bochet et al., 2013) can form tumor-associated fibroblast-like cells. Also, ASCs can be incorporated into blood vessels as pericytes contributing to tumor progression and AT remodeling through the pericyte–fibroblast transition (Zhang et al., 2012; Di Carlo and Peduto, 2018) or adipogenesis (Hosaka et al., 2016). AT-derived pericytes support tumor progression, since an acquired pericytic phenotype was observed in liposarcoma (Shen et al., 2016), and mammary carcinoma cells (Shenoy et al., 2016). The trans-differentiation of mesenchymal breast tumor cells into post-mitotic and functional adipocytes has recently been demonstrated in murine and human breast cancer model *in vivo*, along with its contribution to the inhibition of tumor invasion and metastasis (Ishay-Ronen et al., 2019). These findings demonstrate the importance of the behavior of the healthy and neoplastic cells in AT remodeling, while also suggesting that the pro-adipogenic program activation might be a useful approach in anticancer drug conceptualization.

### Bone Marrow Adipose Tissue and Neoplasms

Bone marrow adipose tissue represents a distinct, heterogenous fat depot with an important role in hematopoiesis, skeletal biology, and tumors, known to provide a supportive microenvironment for hematological malignancies, such as multiple myeloma and leukemias, and a number of solid tumor metastases including melanoma (Chen et al., 2016) and breast and prostate tumors (Zhang et al., 2019). As BMAT expansion during aging interferes with hematopoietic stem/progenitor cell (HSPC) maintenance, BMAT appears as a negative regulator of hematopoiesis (Naveiras et al., 2009; Ambrosi et al., 2017). Nevertheless, a recent study suggested that stem cell growth factor 1, produced by BMAT, supports primitive HSPC survival in mice (Zhou et al., 2017), indicating the complex effects of BMAT on neoplastic hematopoietic cells dependent on the differentiation and disease stage.

Studies using mouse *in vivo* and human *in vitro* models showed that BM adipocytes support the survival, proliferation, and even chemoresistance of malignant cells. Adipokines and free fatty acids released by BM adipocytes directly or indirectly interfere with cells of bone remodeling or hematopoiesis (Hardouin et al., 2016). Tumor cells can induce the phosphorylation of hormone-sensitive lipase and activate lipolysis in BMAT, allowing the transfer of fatty acids from adipocytes to tumor cells in AML (Shafat et al., 2017; Tabe et al., 2020), multiple myeloma (Morris et al., 2020), and prostate tumor (Herroon et al., 2019). Importantly, after chemotherapy, marrow necrosis and the loss of myeloid tissue may result in a BMAT expansion (Hwang and Panicek, 2007; Costa and Reagan, 2019), which might create a favorable niche for tumor cells.

Moreover, metastatic tumor cells interact with medullar adipocytes forming a specific metastatic niche in BM (Morris and Edwards, 2016; Diedrich et al., 2018). BMAT increases during an early stage of murine myeloma, where marrow adipocytes localize along the tumor–bone interface at later stages of the disease. In addition, myeloma cells downregulate BMAT-derived adiponectin, adjusting the marrow microenvironment to support cancer progression (Morris et al., 2020). Myeloma cells also reprogram adipocytes via the increased methylation of PPARγ and altered adipokine production, which activates osteoclastogenesis and osteolytic lesion formation (Liu et al., 2019).

Besides, tumor cells’ survival mechanisms can be activated through the interplay of inflammatory and metabolic pathways in BMAT. As such, metastatic prostate tumor cell-derived IL-1β induced prostaglandin E2 expression and lipolysis in BM adipocytes, coinciding with the pro-survival pathway activation in tumor cells (Herroon et al., 2019). Also, BMAT-induced reactive oxygen species (ROS) production and the heme oxygenase-1 overexpression resulted in the activation of survival molecules survivin (BIRC5) and Bcl-xl in metastatic prostate and breast tumor cells (Diedrich et al., 2018; Herroon et al., 2018). A recent study interestingly suggests that marrow malignancy might affect the adipogenic commitment of resident mesenchymal progenitors, since AML-derived MSCs showed increased adipogenesis, supporting the survival of leukemia progenitor cells (Azadniv et al., 2020).

## Conclusion

Tumor-induced adaptations bring additional complexity to the AT regulation of the healthy and abnormal cell phenotype. Within the spectrum ranging from white to brown adipocytes, the appearance of alternative adipogenic programs in mature adipocytes and their precursors contributes to AT niche remodeling, which occurs in parallel in tumor cells, suggesting the indissolubility of the adipogenic differentiation and tumor progression at the molecular level. The feasibility of cell “trans-differentiation” with the adaptation of tumor cells into adipocyte-like cells, and likely, the reversible conversion of the adipocyte phenotype from the white to beige/brown adipocyte phenotype, point to the possibility for new targets in drug development for oncology patients (Lengyel et al., 2018). Nevertheless, the AT–tumor microenvironment requires accurate comprehension of the time course and stability of various differentiating adipogenic lineages and tumor cell phenotype outcomes.

## Author Contributions

DT and AJ conceived the topic and performed the literature search and wrote the manuscript. SV, IO, TK, and DB revised, co-wrote, and edited the manuscript. All the authors have given their final approval.

## Conflict of Interest

The authors declare that the research was conducted in the absence of any commercial or financial relationships that could be construed as a potential conflict of interest.
